# Evaluating the Efficacy of Secondary Metabolites in Antibiotic-Induced Dysbiosis: A Narrative Review of Preclinical Studies

**DOI:** 10.3390/antibiotics14020138

**Published:** 2025-02-01

**Authors:** Corina Andrei, Anca Zanfirescu, Victor-Pierre Ormeneanu, Simona Negreș

**Affiliations:** Faculty of Pharmacy, “Carol Davila” University of Medicine and Pharmacy, Traian Vuia 6, 020956 Bucharest, Romania; corina.andrei@umfcd.ro (C.A.); victor-pierre.ormeneanu@drd.umfcd.ro (V.-P.O.); simona.negres@umfcd.ro (S.N.)

**Keywords:** antibiotic-induced dysbiosis, polysaccharides, polyphenols, short-chain fatty acid, tight junction proteins, proinflammatory cytokines

## Abstract

Background/Objectives: Drug-induced dysbiosis, particularly from antibiotics, has emerged as a significant contributor to chronic diseases by disrupting gut microbiota composition and function. Plant-derived secondary metabolites, such as polysaccharides, polyphenols, alkaloids, and saponins, show potential in mitigating antibiotic-induced dysbiosis. This review aims to consolidate evidence from preclinical studies on the therapeutic effects of secondary metabolites in restoring gut microbial balance, emphasizing their mechanisms and efficacy. Methods: A narrative review was conducted using PubMed, Scopus, and Web of Science. Studies were selected based on specific inclusion criteria, focusing on animal models treated with secondary metabolites for antibiotic-induced dysbiosis. The search terms included “gut microbiota”, “antibiotics”, and “secondary metabolites”. Data extraction focused on microbial alterations, metabolite-specific effects, and mechanisms of action. Relevant findings were systematically analyzed and summarized. Results: Secondary metabolites demonstrated diverse effects in mitigating the impact of dysbiosis by modulating gut microbial composition, reducing inflammation, and supporting host biological markers. Polysaccharides and polyphenols restored the *Firmicutes/Bacteroidetes* ratio, increased beneficial taxa such as *Lactobacillus* and *Bifidobacterium*, and suppressed pathogenic bacteria like *Escherichia-Shigella*. Metabolites such as triterpenoid saponins enhanced gut barrier integrity by upregulating tight junction proteins, while alkaloids reduced inflammation by modulating proinflammatory cytokines (e.g., TNF-α, IL-1β). These metabolites also improved short-chain fatty acid production, which is crucial for gut and systemic health. While antibiotic-induced dysbiosis was the primary focus, other drug classes (e.g., PPIs, metformin) require further investigation. Conclusions: Plant-derived secondary metabolites show promise in managing antibiotic-induced dysbiosis by restoring microbial balance, reducing inflammation, and improving gut barrier function. Future research should explore their applicability to other types of drug-induced dysbiosis and validate findings in human studies to enhance clinical relevance.

## 1. Introduction

The gut microbiota comprises approximately 100 trillion individual non-pathogenic microorganisms representing diverse species and strains [1,2]. Most biological entities are bacteria, such as *Firmicutes*, *Bacteroidetes*, *Proteobacteria*, *Actinobacteria*, fungi, viruses, protists, and archaea [3,4,5,6,7]. These microorganisms impact immunity, metabolism, and digestion: bacteria, for example, stimulate the production of immunoglobulin A and regulate the activity of T helper and regulatory T cells to maintain immune balance [8,9,10,11,12]. Strains of *Bifidobacterium* and *Lactobacillus* modulate inflammatory processes by balancing anti-inflammatory and proinflammatory cytokines, reducing abnormal inflammatory processes, and lowering the risk of pathogen colonization [13,14,15,16,17]. Additionally, the gut microbiota metabolizes dietary carbohydrates, proteins, fibers, and fats, providing essential nutrients such as short-chain fatty acids (SCFAs) and vitamins B and K [18,19,20]. Thus, the gut microbiota is critical in maintaining immune homeostasis, protecting the body against diseases, and supporting vital processes.

Dysbiosis occurs when the composition, diversity, or function of the gut microbiota is disrupted, leading to an imbalance between beneficial and harmful bacteria [21,22]. This imbalance can result from various factors, including genetic defects, dietary habits, drug administration, stress, and aging [23,24,25,26,27]. Dysbiosis significantly impacts health, being linked to various conditions such as inflammatory bowel diseases (IBDs), metabolic or neurological disorders, and colorectal cancer [28,29,30,31,32,33,34,35,36,37,38]. For instance, a reduction in *Bifidobacterium* levels has been observed in patients with major depressive disorder and autism. *Lactobacillus* levels are elevated in individuals with autism but reduced in depression [31,32]. Similarly, imbalances in *Bacteroides* and *Firmicutes*, key components of the gut microbiota, are associated with IBD, diabetes, and obesity [3,39,40,41,42,43,44]. Drugs are significant contributors to dysbiosis, with antibiotics, metformin, and proton pump inhibitors (PPIs) playing pivotal roles [45,46]. Antibiotics, a cornerstone of modern medicine, often disrupt microbial diversity, reduce SCFA production, hinder metabolic enzyme activity, and promote excessive colonization by antibiotic-resistant pathogens such as *Clostridium difficile* [47,48,49]. Their impact depends on dose, duration, spectrum, and pharmacokinetics [50]. Conversely, metformin enhances SCFA production and alters the microbiota composition, increasing bacteria such as *Enterobacte*riaceae and *Akkermansia muciniphila* [51,52,53]. PPIs cause dysbiosis by increasing gastric pH, which facilitates the overgrowth of certain pathogenic microorganisms [54,55].

Effectively restoring gut microbiota balance is essential to prevent complications of dysbiosis. Current therapeutic interventions include lifestyle modifications, the administration of probiotics, prebiotics, or synbiotics, and fecal microbiota transplantation [56,57,58]. While probiotics have shown clinical benefits in reducing symptoms of dysbiosis, their use is limited by risks such as infections in immunocompromised patients, immune overreactions, and gastrointestinal side effects [59,60,61,62,63,64]. Additionally, challenges such as a lack of regulatory standardization and the inconsistent efficacy of probiotics highlight the need for alternative solutions [16,57,65,66,67].

Natural plant-derived compounds have emerged as promising therapeutic agents for managing dysbiosis [68]. Polyphenols and polysaccharides exhibit antioxidant, anti-inflammatory, antimicrobial, and prebiotic properties [69,70,71,72,73]. These compounds alleviate the downstream effects of dysbiosis by directly impacting host biological markers and/or targeting opportunistic pathogens, mitigating dysbiosis of various etiologies [74,75,76,77]. For example, in preclinical models, polyphenols and alkaloids restore the *Firmicutes*/*Bacteroidetes* ratio disrupted by high-fat diets and enhance gut barrier integrity and metabolic parameters, including insulin sensitivity [78,79,80,81,82,83,84,85,86,87].

In animal models of colitis, polyphenols and polysaccharides reduce inflammation by suppressing proinflammatory cytokines such as tumor necrosis factor (TNF)-α and interleukin (IL)-1β, increase *Lactobacillus* abundance, and decrease that of *Bacteroides* [88,89,90]. Furthermore, their reduced absorption prolongs their contact with the intestinal mucosa, enhancing the therapeutic effects [91]. These findings suggest that phytochemicals are effective in managing the dysbiosis of various etiologies.

Despite growing evidence of the benefits of plant-derived secondary metabolites, no comprehensive review has summarized their effects on drug-induced dysbiosis. Research in this field remains limited, with most studies concentrating on antibiotic-induced dysbiosis. This manuscript addresses this gap by analyzing preclinical studies on the effects of plant-derived secondary metabolites in counteracting antibiotic-induced dysbiosis and the underlying mechanisms. By examining these mechanisms, this review aims to enhance our understanding of potential therapeutic strategies for restoring microbial homeostasis in patients undergoing long-term drug treatments.

## 2. Prominent Microbial Species in Drug-Induced Dysbiosis

Antibiotics are the primary drugs associated with drug-induced dysbiosis. Tetracyclines significantly reduce the diversity and abundance of *Bifidobacterium* species, key anaerobic bacteria essential for gut health [92]. Depending on individual resilience, recovery can take weeks to months [93,94,95]. Clarithromycin decreases *Enterobacteria* (e.g., *Escherichia* and *Shigella*), as well as *Bifidobacterium* and *Lactobacillus* species, which are crucial gut ecosystem stability, with effects persisting for up to five weeks [92,96]. Macrolides also increase *Proteobacteria*, including opportunistic pathogens, potentially contributing to dysbiosis [97].

Broad-spectrum penicillins, such as amoxicillin, decrease *Bacteroides*, *Bifidobacterium*, and *Lactobacillus* species while transiently promoting the overgrowth of *Escherichia coli* and other *Enterobacteriaceae* (e.g., *Klebsiella* and *Enterobacter*). Their effects on anaerobic bacteria, including *Clostridium* species, are minimal, with normalization typically occurring within two to three weeks [98,99,100]. Amoxicillin-clavulanic acid further reduces *Bifidobacterium* species, particularly *Bifidobacterium adolescentis* and *Bifidobacterium bifidum*, as well as *Lachnospiraceae* and *Ruminococcaceae*, essential groups involved in fiber degradation and SCFA production [101]. Antibiotic-resistant *Enterobacteriaceae* often proliferate during treatment [102]. Conversely, phenoxymethylpenicillin and nitrofurantoin have minimal impacts on gut microbiota diversity and abundance [97,103,104,105].

Broad-spectrum antibiotics often reduce beneficial bacteria, such as *Faecalibacterium prausnitzii*, *Bifidobacterium longum*, and *Roseburia*, while promoting the growth of pathogenic species, including *Escherichia/Shigella* and *Klebsiella pneumoniae*. The recovery of *Bifidobacterium* and *Lactobacillus* can take several weeks to months, though resistant species and dysbiotic effects may persist for longer. For macrolides, an altered microbiota composition can last six months or more [97,106,107]. Reduced alpha diversity is linked to increased opportunistic pathogens during antibiotic treatment [44,108].

While antibiotics are well-documented as major disruptors of microbial diversity and balance, increasing evidence suggests that other commonly used drugs such as PPIs, metformin, opioids, and antipsychotics can also induce significant changes in gut flora [46,52,53,109,110,111,112,113,114,115,116,117,118,119,120,121,122,123,124,125,126,127,128,129,130,131,132,133,134,135,136,137,138,139,140,141,142,143,144,145,146,147,148,149,150,151,152,153,154,155]. These alterations, collectively referred to as drug-induced dysbiosis, are associated with shifts in the abundance and diversity of specific microbial species (Table 1), potentially leading to adverse health outcomes.

**Table 1:** Prominent microbial species affected by drug-induced dysbiosis.

Drug Category	Effects on Gut Microbiota	Commonly Affected Taxa	Recovery Timeframe
Antibiotics	Reduction in diversity and abundance of beneficial bacteria; proliferation of opportunistic/pathogenic species	↓ *Bifidobacterium*, *Lactobacillus*, *Faecalibacterium prausnitzii*, *Roseburia* ↑ *Escherichia/Shigella*, *Klebsiella pneumoniae*, *Proteobacteria*	Weeks to months depending on antibiotic type and individual resilience
PPIs	Shift toward pathogenic species, reduced protective bacteria, and overall decreased diversity	↑ *Bifidobacterium* [109], *Streptococcus* [110], *Rothia mucilaginosa* [111], *Streptococcus anginosus* [112], *Enterobacter*, *Klebsiella*, *Citrobacter* [115,116] *↓ Clostridiales*, *Clostridiaceae*, *Lachnospiraceae*, *Ruminococcaceae* [115,116]	Variable; changes often persist during PPI use.
Metformin	Reduced diversity but increase in beneficial SCFA-producing bacteria and reduction in harmful taxa	↑ *Akkermansia muciniphila*, *Roseburia*, *Butyricimonas* ↓ *Clostridia*, *Peptostreptococcaceae* [42,47,50]	Diversity reduction after 2–3 doses; partial recovery in 7 days
Opioids	Disruption in gut integrity, reduced motility, and systemic inflammation leading to pathogenic overgrowth	↑ *Enterococcaceae*, *Peptostreptococcaceae*, *Streptococcaceae*, *Collinsella*, *Prevotella*, *Klebsiella*, *Veillonella* [134,156,157,158] ↓ *Ruminococcaceae*, *Lachnospiraceae*, *Bifidobacterium sp.*, *Akkermansia muciniphila* [134,156,158]	Chronic disruption with long-term use
Antipsychotics	Heterogeneous effects; reduced α-diversity with increases in pathogenic and fermentative taxa	↑ *Klebsiella*, *Escherichia/Shigella*, *Streptococcaceae*, *Erysipelotrichaceae* [140,142,143,147,152,153] *Bacteroides*, *Roseburia* [139,142,145,153,155]	Varies by medication type and patient condition

Legend: ↑, increased; ↓, decreased.

**Figure 1:**
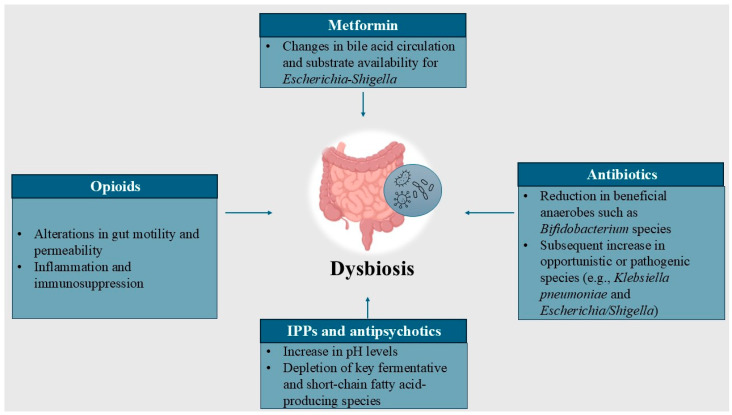
Mechanisms and specific microbial alterations induced by various classes of drugs.

Not only do drug-induced microbial alterations vary significantly across drug classes, but their underlying mechanisms also differ (Figure 1).

Broad-spectrum antibiotics consistently reduce beneficial bacteria (*Bifidobacterium*, *Faecalibacterium*, *Lactobacillus*) while promoting the proliferation of opportunistic pathogens (*Escherichia coli*, *Klebsiella*, *Shigella*), with recovery timelines varying significantly. PPIs and antipsychotics exacerbate dysbiosis by increasing pathogenic taxa and depleting key fermentative and short-chain fatty acid-producing species, heightening risks of systemic and gut-related complications. Metformin, while reducing overall diversity, paradoxically fosters growth in *Akkermansia* and SCFA-producing bacteria, contributing to its therapeutic benefits in diabetes. Opioids further disrupt microbiota via slowed motility and systemic inflammation, favoring pathogenic overprotective species.

Clinically, by inducing dysbiosis, antibiotics carry the risk of resistant strain development, PPIs increase susceptibility to enteric infections, opioids elevate the risk of nosocomial infections, and antipsychotics are associated with metabolic disturbances and weight gain.

## 3. Secondary Metabolites Effective Against Antibiotic-Induced Dysbiosis in Animal Studies

Secondary metabolites have shown significant potential in mitigating antibiotic-induced dysbiosis, with preclinical animal studies highlighting their ability to restore microbial balance, enhance gut barrier integrity, and modulate immune responses (Table 2).

Polysaccharides from various sources consistently demonstrate their ability to reduce dysbiosis by restoring the *Firmicutes/Bacteroidetes* ratio, a key indicator of gut microbial health [159,160,161]. Restoring the F/B ratio helps re-establish microbial diversity promotes the functional recovery of the gut microbiota, including enhanced SCFA production, improved gut barrier integrity, and modulation of immune responses. These outcomes are essential for mitigating the adverse effects of dysbiosis and maintaining overall gut and systemic health. They destroy pathogenic bacteria such as *Proteobacteria*, *Enterococcus*, and *Escherichia-Shigella*, subsequently promoting the growth of beneficial genera such as *Lactobacillus*, *Butyricicoccus*, and *Akkermansia* [161,162]. Polyphenols, such as catechins, quercetin, and baicalin, effectively mitigate dysbiosis by restoring microbial diversity and composition toward normal levels. They increase the relative abundance of beneficial genera like *Lachnospiraceae*, *Ruminococcaceae*, and *Akkermansia* while reducing inflammatory taxa such as *Anaeroplasma* in a dose-dependent manner [163]. Triterpenoid saponins, such as ginsenoside Rk3, also restore the *Firmicutes/Bacteroidetes* ratio by reducing *Firmicutes* and increasing *Bacteroidetes* while simultaneously promoting the growth of beneficial genera such as *Anaerostipes*, *Alloprevotella*, and *Bacteroides* [164]. Glycosides, such as salidroside, also show significant potential in addressing dysbiosis. They promote the recovery of beneficial bacteria, including *Bacteroides*, *Lactobacillus*, and *Bifidobacterium*, while reducing harmful bacteria, such as *Helicobacter* and *Ruminococcus_torques_group*. Salidroside’s effects are dose-dependent, highlighting its tailored application in restoring microbiota balance [165].

Alkaloids, such as those derived from Corydalis saxicola, exhibit targeted restoration of specific bacterial populations. For example, they completely restored *Blautia* to control levels while partially restoring other genera, such as *Clostridium sensu stricto 1* and *Hungatella* [166]. Sesquiterpenoids, like atractylenolide I, effectively increase the abundance of beneficial bacteria, such as *Lactobacillus* and *Bacteroides*, while reducing harmful taxa, such as *Escherichia* and *Candidatus*, with dose-dependent effects [167]. Preclinical studies assessing the effects of these secondary metabolites in antibiotic-induced dysbiosis are summarized in Table 2. These animal models mostly use BALB/c and C57BL/6 mice as well as Sprague-Dawley and Wistar rats as they have a well-established use in studying gut microbiota alterations. Antibiotics like lincomycin, ceftriaxone, and ciprofloxacin were used to induce dysbiosis, mimicking real-world scenarios of antibiotic-induced microbial imbalances. These models allowed researchers to assess how secondary metabolites influence microbial diversity, SCFA production, and gut barrier integrity.

Besides restoring microbiota, these compounds possess other effects that mitigate antibiotic-induced dysbiosis.

Polysaccharides from sources such as Dictyophora indusiate, Deglet Noor, Ganoderma lucidum, and Poria cocos [160,168,169], polyphenols, and alkaloids upregulate the expression of tight junction proteins such as zonulin-1 and occludin [163,164], improving gut barrier integrity. These compounds exhibit significant anti-inflammatory effects by reducing proinflammatory cytokines, including TNF-α, IL-1β, and IL-6. Polysaccharides are particularly effective in mitigating dysbiosis-induced inflammation [160,163]. Polyphenols and triterpenoid saponins also contribute by lowering TNF-α and IL-1β, while saponins promote anti-inflammatory cytokines like IL-10, ensuring a balanced immune response [164]. Alkaloids similarly reduce lipopolysaccharides and cytokines such as IL-1β, alleviating inflammation [167].

Polysaccharides [169,170], polyphenols [163], and salidroside [165] enhance SCFA production, increasing the levels of butyrate, propionate, valeric acid, and acetic acid, which support gut health, homeostasis, and energy metabolism. Additionally, alkaloids influence critical metabolic pathways, including bile acid and amino acid metabolism, further contributing to the restoration of gut health [166].

Overall, the tested compounds consistently demonstrate their ability to restore the gut microbiota, suppress proinflammatory responses, enhance gut barrier function, and promote SCFA production across various chemical classes. These multifaceted effects make them promising candidates for mitigating antibiotic-induced dysbiosis and its associated complications.

**Table 2:** Preclinical studies assessing the effects of secondary plant metabolites on the microbiota of animals with antibiotic-induced dysbiosis.

Chemical Class	Plant/Active Ingredient	Dysbiosis-Inducing Antibiotic Treatment	Animal	Effects on the Microbiota	Author
Polysaccharides	Polysaccharides from Dictyophora indusiate	Clindamycin + metronidazole	BALB/c mice	restored the decreased *Firmicutes/Bacteroidetes* ratio; ↓ *Proteobacteria*, *Enterococcus*, and *Bacteroides*; ↑ *Lactobacillaceae* and *Ruminococcaceae*.	[161]
	Lentinan	Antibiotic cocktail (vancomycin + neomycin sulfate + metronidazole+ ampicillin)	C57BL/6 J mice	efficiently restored the ratio *Firmicutes/Bacteroidetes*; ↓ *Parabacteroides*, *Klebsiella*, *Akkermansia*, *Proteobacteria*,and *Actinobacteria*; ↑ *Oscillospira*.	[160]
	Polysaccharides from Deglet Noor dates	Amoxicylin	BALB/c mice	↑ *Lachnospiraceae_NK4A136* and *Ruminococcaceae*; ↓*Bacteroides*, *Klebsiella*, *Escherichia-Shigella*, *Proteobacteria*, and *Enterococcus*.	[171]
	Polysaccharides from Fagopyrum esculentum Moench bee pollen	Ceftriaxone	BALB/c mice	restored *Bacteroidetes/Firmicutes* ratio; ↑ *Lachnospiraceae_NK4A136_group*, *uncultured_bacterium_f_Lachnospiraceae*, and *Akkermansia*; ↓ *Bacteroides*; did not reduce the abundance of *Staphylococcus*.	[159]
	Polysaccharides from Panax ginseng	Lincomycin hydrochloride	Balb/c mice	↑*Firmicutes*, *Lactobacillus*, *Lactococcus*, and *Streptococcus*; ↓ *Bacteroidetes*, *Proteobacteria* and *Actinobacteria*.	[172]
	Polysaccharides from Panax quinquefolius	Lincomycin hydrochloride	Wistar rats	↑ *Lactobacillus* and *Bacteroides*; ↓ *Blautia* and *Coprococcus*.	[173]
	Polysaccharides from Schisandra chinensis	Lincomycin hydrochloride	Wistar rats	↑ *Blautia*, *Intestinibacter* and *Lachnospiraceae-UCG-008*; ↓ *Ruminococcus-1*, *Ruminococcaceae-UCG-014*, and *Erysipelatoclostridium.*	[174]
	Polysaccharides from Astragalus membranaceus	Lincomycin hydrochloride	Wistar rats	↑ *Bacteroidetes* and *Proteobacteria*; ↓ *Firmicutes*, *Pseudomonas*, *Allobaculum*, and *Coprococcus*.	[175,176]
	Polysaccharides from Ganoderma lucidum	Antibiotic cocktail (vancomycin + neomycin sulfate + metronidazole+ ampicillin)	C57BL/6 J mice	↓ *Firmicutes*/*Bacteroidetes* ratio; ↑ *Lactobacillus* and *Roseburia*.	[168]
	Polysaccharides from bamboo shoot (Leleba oldhami Nakal)	Lincomycin hydrochloride	Kunming mice	↑ *Bacteroides*, *Lactobacillus*, and *Lachnospiraceae_NK4A136_group*; ↓ *Escherichia-Shigella*, *Ruminiclostridium_9*, *Ruminococcus_1*, *Proteus* and *Firmicutes*/*Bacteroidetes* ratio.	[162]
	Fucoidan	Ciprofloxacin + metronidazole	C57BL/6J mice	↑ *Ruminococcaceae_UCG_014* and *Akkermansia*; ↓ *Proteus* and *Enterococcus*.	[177,178]
	Exopolysaccharides from Antrodia cinnamomea	Lincomycin hydrochloride	ICR mice	↑ *Ligilactobacillus*, *Lactobacillus*, *Roseburia*, *Alistipes*, *Parabacteroides*, and *Lachnospiraceae_NK4A136* *_group*; ↓ *Enterococcus*, *Staphylococcus*, and *Shigella*, in a dose-dependent manner.	[179]
	Polysaccharides from purple sweet potato	Lincomycin hydrochloride	BALB/C mice	↑ *Muribaculaceae* and *Bacteroidaceae*; ↓ *Enterobacteriaceae* family (such as *Escherichia coli* and *Klebsiella*).	[170]
	Inulin-type fructans	Antibiotic cocktail (vancomycin + neomycin sulfate + metronidazole+ ampicillin)	BALB/c mice	did not promote the recovery of microbial community composition.	[180]
	Long-chain inulin	Antibiotic cocktail (vancomycin + neomycin sulfate + metronidazole+ ampicillin)	BALB/c mice	did not restore gut microbiota composition at the genus level.	[180]
	Pectins	Antibiotic cocktail (vancomycin + neomycin sulfate + metronidazole+ ampicillin)	C57BL/6J mice	↑ *Bifidobacterium*, *Faecalibaculum*, *Ruminococcus_torques_group*, *Clostridium_innocuum_group*, and *Enterorhabdus*; ↓ *Alistipes* and *Mucispirillum*.	[181]
	Polysaccharides from Poria cocos	Antibiotic cocktail (vancomycin + neomycin sulfate + metronidazole+ ampicillin)	C57BL/6N mice	↑ *Bacillota*, *Allobaculum*, *Ruminococcus*, and *Turicibacter*; ↓ *Verrucomicrobiota*, *Pseudomonadota*, *Parabacteroides*, *Akkermansia*, *Desulfovibrio*, *Clostridium*, and *Sutterella*	[182]
	Polysaccharides from Hericium erinaceus	Lincomycin hydrochloride	Wistar rats	↑ *Lactobacillus* and *Butyricicoccus*; ↓ *Enterococcus* and *Allobaculum.*	[169]
Polyphenols	Green tea polyphenols (catechins)	Cefixime	Kunming mice	↑ *Bacteroidetes*, *Verrucomicrobia*, *Lachnospiraceae_NK4A136_group*, *Ruminococcaceae_UCG-014*, *Ruminiclostridium-5*, and *Akkermansia norank_f_Muribaculaceae*; ↓ *Firmicutes*, *Epsilonbacteraeota* and *Anaeroplasma*; !low-dose catechins significantly increased the relative abundance of *Lactobacillus* while middle- or high-dose cathechins significantly decreased it.	[163]
	Baicalin	Lyncomicin	Piglets	↓ *Staphylococcus*, *Dolosicoccus*, *Escherichia-Shigella*, and *Raoultella*. ↑ *norank_f_Muribaculaceae*, *Prevotella*, and *Akkermansia*.	[183,184]
	Quercetin	Amoxicillin/clavulanate potassium	Sprague-Dawley rats	↑ *Lactobacillus*, *Bifidobacterium*, and Bacteroides; ↓ *Clostridium* and *Enterobacteriaceae*.	[185,186]
	Nobiletin	Cefuroxime + levofloxacin	C57BL/6 mice	↑ *f_Lachnospiraceae*; Prevents increase in Escherichia coli and Shigella.	[187]
Glycosides	Salidroside	Ceftriaxone	C57BL/6J mice	↑*Bacteroides*, *Parabacteroides*, *Lactobacillus*, and *Bifidobacterium*, *Dubosiella*; ↓ *norank_f_Muribaculaceae*, *Helicobacter*, and *Ruminococcus_torques_group*.These effects were dose-dependent.	[165]
Triterpenoids and saponins	Ginsenoside Rk3	Lincomycin	C57BL/6J mice	↓ *Firmicutes*. ↑ *Bacillaceae*, *Bacteroidaceae*, *Prevotellaceae*, *Anaerostipes*, *Alloprevotella*, *Lachnoclostridium*, *Blautia*, and *Bacteroidetes*.	[164]
	Ginsenoside Rh4	Lincomycin	C57BL/6 mice	↑ *Bacteroides*, *Alloprevotella*, *Blautia*, and *Parabacteroides*; ↓ *Serratia*, *Allobaculum*, *Hungatella*, and the *Firmicutes/Bacteroidetes* ratio.	[188]
	Protopanaxatriol	Cephalosporin	C57BL/6 mice	↑ *Firmicutes*, *Hydrogenophaga*, *Mesorhizobium*, and *Sphingomonas*; ↓ *Christensenella* and *Bacteroidetes*.	[189]
	Ursolic acid	Chlortetracycline	Kunming mice	↑ *Lactobacillus*; ↓ *Enterobacteriaceae* and *Proteobacteria.*	[190]
Alkaloids	Alkaloids from Corydalis Saxicola	Imipenem/cilastatin sodium	Sprague–Dawley rats	↑ *Blautia* and to a smaller extent *Clostridium sensu stricto 1*; ↓ *Hungatella* and *Intestinibacter.*	[165]
Sesquiterpenoids	Atractylenolide I	Antibiotic cocktail (vancomycin + neomycin sulfate + metronidazole+ ampicillin)	C57BL/6 mice	↑ *Lactobacillus* and *Bacteroides*; ↓ *Escherichia* and *Candidatus* in a dose-dependent manner.	[165]

Legend: ↑, increased; ↓, decreased.

## 4. Discussion

The findings of this review underscore the potential of secondary metabolites, including polysaccharides, polyphenols, alkaloids, and triterpenoid saponins, in reducing the effects of drug-induced dysbiosis by directly killing opportunistic pathogens while enabling the recovery of host biological markers. These include

- Increasing gut barrier integrity: strengthening the intestinal barrier by upregulating tight junction proteins such as zonula occludens-1 and occludin maintains gut permeability and prevents harmful substances from entering the bloodstream;
- Reducing inflammation: reducing proinflammatory cytokines like TNF-α, IL-1β, and IL-6 while increasing anti-inflammatory cytokines such as IL-10 to restore immune balance;
- Enhancing the production of SCFAs such as butyrate, propionate, and acetate, which are essential for gut health and energy metabolism and reduce systemic inflammation;
- Regulating metabolic markers: modulating bile acid metabolism and metabolites that impact energy homeostasis and glucose sensitivity.

**Figure 2:**
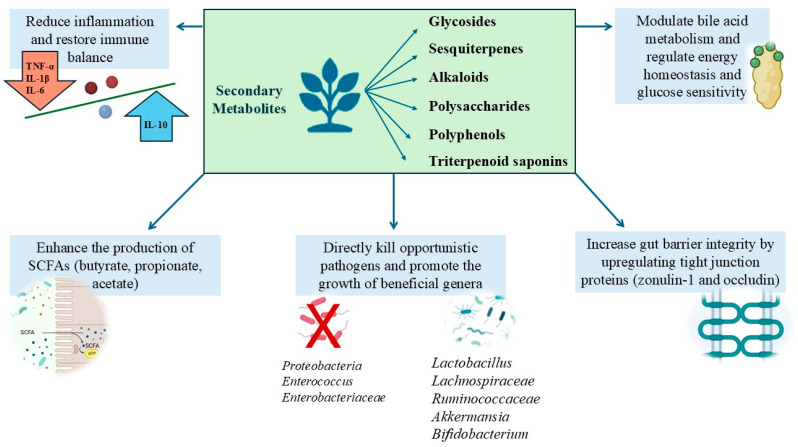
Mechanisms of plant-derived secondary metabolites in antibiotic-induced dysbiosis.

In essence, these secondary metabolites promote functional and physiological recovery of the host, emphasizing systemic health benefits rather than simply restoring the composition of gut microbiota (Figure 2).

Plant-derived secondary metabolites indirectly enhance SCFA production by selectively promoting the growth of SCFA-producing bacterial strains, such as *Akkermansia muciniphila*, *Lachnospiraceae*, and *Ruminococcaceae*. These metabolites provide substrates and create favorable conditions for beneficial bacteria, resulting in increased levels of SCFAs such as butyrate, propionate, and acetate. Furthermore, these bacterial groups modulate bile acid metabolism and promote intestinal integrity [191].

Additionally, they restore the *Firmicutes/Bacteroidetes* ratio, a key marker of gut microbial health associated with metabolic disorders, obesity, and inflammatory diseases. Beneficial bacteria such as *Lactobacillus* and *Bifidobacterium* strengthen gut barrier integrity, support SCFA production, and modulate immune responses while suppressing pathogenic taxa like *Escherichia-Shigella* to reduce inflammatory processes and prevent opportunistic infections. These findings highlight the potential of secondary metabolites to restore gut microbial homeostasis and alleviate dysbiosis-related conditions.

Specific bacterial contributions include

- *Lactobacillus*: modulates gut health, immunity, and metabolic pathways. Strains like *L. rhamnosus* and *L. reuteri* enhance the production of regulatory T cells and increase bacterial diversity, reducing systemic inflammation [192]. Additionally, *Lactobacillus* intervention alleviates colitis by interacting with other beneficial bacteria like *Akkermansia*, leading to improved immune regulation and intestinal health [193];
- *Bifidobacterium*: produces acetate and propionate, reduces gut inflammation, improves the integrity of the gut barrier, and inhibits harmful taxa like *Escherichia coli* and *Clostridium* while promoting beneficial groups such as *Akkermansia* and *Lachnospiraceae* [194];
- *Akkermansia muciniphila*: fortifies the intestinal mucus layer, reduces gut permeability, and modulates immune responses, particularly in metabolic diseases such as obesity and type 2 diabetes [193,195];
- *Roseburia:* butyrate-producing bacteria that maintain the intestinal epithelial barrier and reduce inflammatory cytokine production [196]. Decreased *Roseburia* abundance is linked to IBD, metabolic disorders, and neurodegenerative diseases like Parkinson’s disease [197,198];
- *Lachnospiraceae*: support fiber metabolism, SCFA production, and gut barrier integrity while modulating inflammation and reducing systemic toxin levels [199,200];
- *Ruminococcaceae*: produce SCFA supporting fiber fermentation and energy metabolism, reduce inflammation, improve glucose metabolism, and strengthen intestinal barrier function [199].

Simultaneously, these metabolites reduce proinflammatory bacteria such as *Peptostreptococcaceae* and *Clostridium sensu stricto 1* associated with infections and dysbiosis. However, some taxa, such as *Ruminococcus torques*, may have context-specific roles. While their reduction is beneficial in antibiotic-induced dysbiosis, their depletion in healthy individuals could have unintended consequences, highlighting the need for precise targeting of microbial restoration to avoid unintended consequences.

Microbiota restoration translates into systemic health improvements, such as reduced inflammation, enhanced immunity, or better metabolic outcomes.

Current evidence primarily focuses on antibiotic-induced dysbiosis, leaving gaps in understanding the efficacy of secondary metabolites against dysbiosis caused by other drugs (e.g., PPIs, opioids, and antipsychotics). Therefore, there is an urgent need for comparative studies to verify whether the mechanisms demonstrated in antibiotic-induced dysbiosis apply broadly to other drug classes. Additionally, most findings derive from animal studies, and their applicability to humans remains uncertain.

The effects of several plant-derived metabolites on human microbiota are well-known. For example, non-digestible carbohydrates (e.g., resistant starch, fructooligosaccharides) promote beneficial bacteria like *Lactobacillu*s, *Ruminococcus*, *E. rectale*, and *Roseburia* [201], while fructooligosaccharides increase *Bifidobacterium*, promoting butyrate production. Similarly, plant-based fats improve gut health by increasing the *Bacteroidetes/Firmicutes* ratio and *Bifidobacterium* abundance. Omega-3 fatty acids enhance beneficial bacteria like *Lactobacillus* and *Akkermansia muciniphila*. Polyphenols from tea, fruits, and vegetables were reported to increase *Bifidobacterium* and *Lactobacillus*, boosting SCFA production and reducing inflammation [202]. However, we identified no clinical trial assessing the effectiveness of plant-derived metabolites in drug-induced dysbiosis. Furthermore, differences in microbiome composition between species, dosage, and metabolite bioavailability may influence outcomes. Human studies are urgently needed to validate the efficacy and safety of these secondary metabolites across diverse populations and drug-induced dysbiosis types.

Most studies assess individual secondary metabolites, neglecting the potential synergistic or antagonistic interactions when multiple metabolites are combined or administered alongside conventional therapies. Developing standardized protocols for dysbiosis induction, metabolite dosing, and microbiota analysis would improve the comparability of findings and optimize existing therapeutic strategies.

## 5. Materials and Methods

This narrative review was designed to summarize the recent literature on plant secondary metabolites such as polysaccharides, polyphenols, alkaloids, triterpenoid saponins, and glycosides, which mitigate antibiotic-induced dysbiosis.

A systematic literature search was conducted across PubMed, Web of Science, and Scopus to retrieve all articles published on this theme. The following search terms and Boolean operators were employed: “gut microbiota” OR “gut microbiome” OR “dysbiosis” OR “microbial diversity” OR “microbial composition” AND “antibiotic*” OR “proton pump inhibitors” OR “metformin” OR “opioid*” OR “antipsychotic*”.

The inclusion criteria comprised studies (a) addressing microbial alterations induced by drugs and interventions involving plant secondary metabolites and (b) published in peer-reviewed journals. The following were excluded: (a) articles not written in English; (b) studies focusing on non-drug-induced dysbiosis (e.g., diet-induced dysbiosis) without addressing drug effects; and (c) reviews, commentaries, or meta-analyses not reporting new data.

Two independent reviewers screened the articles in three stages: title, abstract, and full text. The following data were extracted from each study:

▪ Study characteristics: animal strain, antibiotic utilized to treat dysbiosis;
▪ Drug information: type of drug treatment used to induce dysbiosis;
▪ Intervention information: type of secondary metabolite, induced microbial alterations.

No ethical approval was required as this is a narrative review based on the published literature.

## 6. Conclusions

This review highlights the promising therapeutic potential of plant-derived secondary metabolites in mitigating antibiotic-induced dysbiosis by restoring microbial balance, reducing inflammation, and enhancing gut barrier integrity. While these metabolites might address dysbiosis caused by other drugs, such as PPIs, metformin, and opioids, research in these areas is limited and warrants further exploration.

However, the findings are predominantly based on preclinical studies, and their translation to human applications remains uncertain due to differences in microbiota composition, metabolite bioavailability, and dosing across species. Bridging this gap requires well-designed clinical trials to validate the efficacy, safety, and applicability of these secondary metabolites in human populations. Future research should also explore combination therapies involving secondary metabolites, probiotics, and conventional treatments to optimize therapeutic outcomes. These efforts can pave the way for integrative strategies to manage drug-induced dysbiosis and improve overall gut health.

## Data Availability

All data generated or analyzed during this study are included in this published article.
